# The Current Evidence on the Association Between the Urinary Microbiome and Urinary Incontinence in Women

**DOI:** 10.3389/fcimb.2019.00133

**Published:** 2019-05-01

**Authors:** Yashini Govender, Iwona Gabriel, Vatche Minassian, Raina Fichorova

**Affiliations:** ^1^Department of Obstetrics, Gynecology and Reproductive Biology, Harvard Medical School, Brigham and Women's Hospital, Boston, MA, United States; ^2^Division of Urogynecology, Brigham and Women's Hospital, Boston, MA, United States

**Keywords:** metagenomics, culturable bacteria, urinary tract infection, stress incontinence, urgency incontinence, mixed incontinence, urinary microbiome, microbiota

## Abstract

Urinary incontinence (UI) is a burdensome condition with high prevalence in middle-aged to older women and an unclear etiology. Advances in our understanding of host-microbe interactions in the urogenital tract have stimulated interest in the urinary microbiome. DNA sequencing and enhanced urine culture suggest that similarly to other mucosal sites, the urinary bladder of healthy individuals harbors resident microbial communities that may play distinct roles in bladder function. This review focused on the urobiome (expanded quantitative urine culture-based or genomic sequencing-based urinary microbiome) associated with different subtypes of UI, including stress, urgency and mixed urinary incontinence, and related syndromes, such as interstitial cystitis and overactive bladder in women, contrasted to urinary tract infections. Furthermore, we examined clinical evidence for the association of the urinary microbiome with responses to pharmacotherapy for amelioration of UI symptoms. Although published studies are still relatively limited in number, study design and sample size, cumulative evidence suggests that certain *Lactobacillus* species may play a role in maintaining a healthy bladder milieu. Higher bacterial diversity in the absence of *Lactobacillus* dominance was associated with urgency UI and resistance to anticholinergic treatment for this condition. UI may also facilitate the persistence of uropathogens following antibiotic treatment, which in turn can alter the commensal/potentially beneficial microbial communities. Risk factors of UI, including age, menopausal status, sex steroid hormones, and body mass index may also impact the urinary microbiome. However, it is yet unclear whether the effects of these risks factors on UI are mediated by urinary host-microbe interactions and a mechanistic link with the female urogenital microbiome is still to be established. Strategies for future research are suggested.

## Introduction

Recently, there has been a great interest in the impact of the human microbiome on urinary tract diseases and disorders. Previous reviews on this topic addressed methodological advances in the urinary microbiome field and the clinical implications of the microbiome in urinary tract diseases (Angelini, 2017; Brubaker and Wolfe, 2017; Drake et al., 2017; Hiergeist and Gessner, 2017; Mueller et al., 2017; Thomas-White et al., 2017; Wolfe and Brubaker, 2018). This review has a specific focus on the urinary incontinence disorder, including challenges to differential diagnosis, therapy and risk factors, in association with the female urinary microbiome. The review also highlights strategies used by vaginal and gut microbiome studies that could inform future microbiome explorations in this field.

### Definitions and Risk Factors of the Urinary Incontinence (UI) Disorder and Lower Urinary Tract Syndromes With Overlapping Symptoms

Urinary incontinence (UI) is a burdensome lower urinary tract disorder of involuntary void of urine, urinary frequency and nocturia, common among women of all ages, with a prevalence ranging from 30 to 60% in middle-aged and older women (Landefeld et al., 2008). UI is generally classified into three main subtypes including stress (SUI), urgency (UUI), and mixed UI (MUI). SUI is the complaint of urine loss associated with exertion such as cough, sneeze, lift, or laugh; UUI is the complaint of urine loss associated with urgency; finally, women who have co-existing symptoms of stress and urgency UI are defined as MUI (Haylen et al., 2010). While several UI risk factors are known, including menopausal status, age, body mass index (BMI), and parity, the pathophysiology of UI, and particularly that of UUI and MUI, remains poorly defined. Moreover, it is not fully understood how to predict new onset UI, prevent progression of UI, and ultimately how to promote lower urinary tract health.

Several urinary tract disorders have symptoms overlapping with UI and at the same time can coincide with UI, creating challenges in differential diagnosis but also in differential assessment of risk factors. Those include urinary tract infections (UTIs), overactive bladder syndrome (OAB), and interstitial cystitis (IC). Confirmation of symptomatic UTI, is typically based on standard urine culture, detecting uropathogenic bacteria ≥10^5^ CFU/ml in a voided urine specimen or ≥10^2^ CFU/ml in catheterized or suprapubic aspirated urine samples (Wilson and Gaido, 2004). However, this standard methodology has a high false negative rate and can result in underreporting of uropathogens (Kline and Lewis, 2016; Price et al., 2016, 2018). OAB is a syndrome encompassing symptoms of urinary urgency, usually accompanied by frequency and nocturia, with or without UUI (Haylen et al., 2010). IC is characterized by suprapubic pain, dysuria in addition to urinary frequency, urgency, and nocturia in the absence of UTIs (Abernethy et al., 2017).

The mechanisms underlying clinical presentation similarities between the urinary tract disorders that present with urge for urination and incontinence is unknown. This review presents evidence that underlying pathophysiology may involve altered urinary microbiota defined here as the sum of microorganisms residing in the urinary tract anatomical niche in health and disease. The search is on for culture-based and genomic sequence-based microbiome characteristics and metagenomic signatures of the urinary microbiota (defined here as urobiome) in order to better understand and differentially diagnose UI and manage the urinary tract disorders.

### Detection of Urinary Microbiota and Microbiome of the Urinary Tract

Urine within the healthy urinary tract has long been considered sterile based on limited culture techniques available in the past. However, this old dogma has been challenged after the application of culture-independent 16S rRNA gene sequencing and expanded quantitative urine culture (EQUC) with mass spectrometry (MALDI-TOF) (Hilt et al., 2014). These technological advances have allowed the detection of microbes not just in voided urine (Siddiqui et al., 2011; Fouts et al., 2012; Wolfe et al., 2012; Lewis et al., 2013) which may carry contaminants from outside the urinary tracts e.g., the vulvovaginal area, but also in catheterized urine (Fouts et al., 2012; Wolfe et al., 2012) and suprapubic bladder aspirates (Wolfe et al., 2012) from asymptomatic individuals.

**Figure 1:**
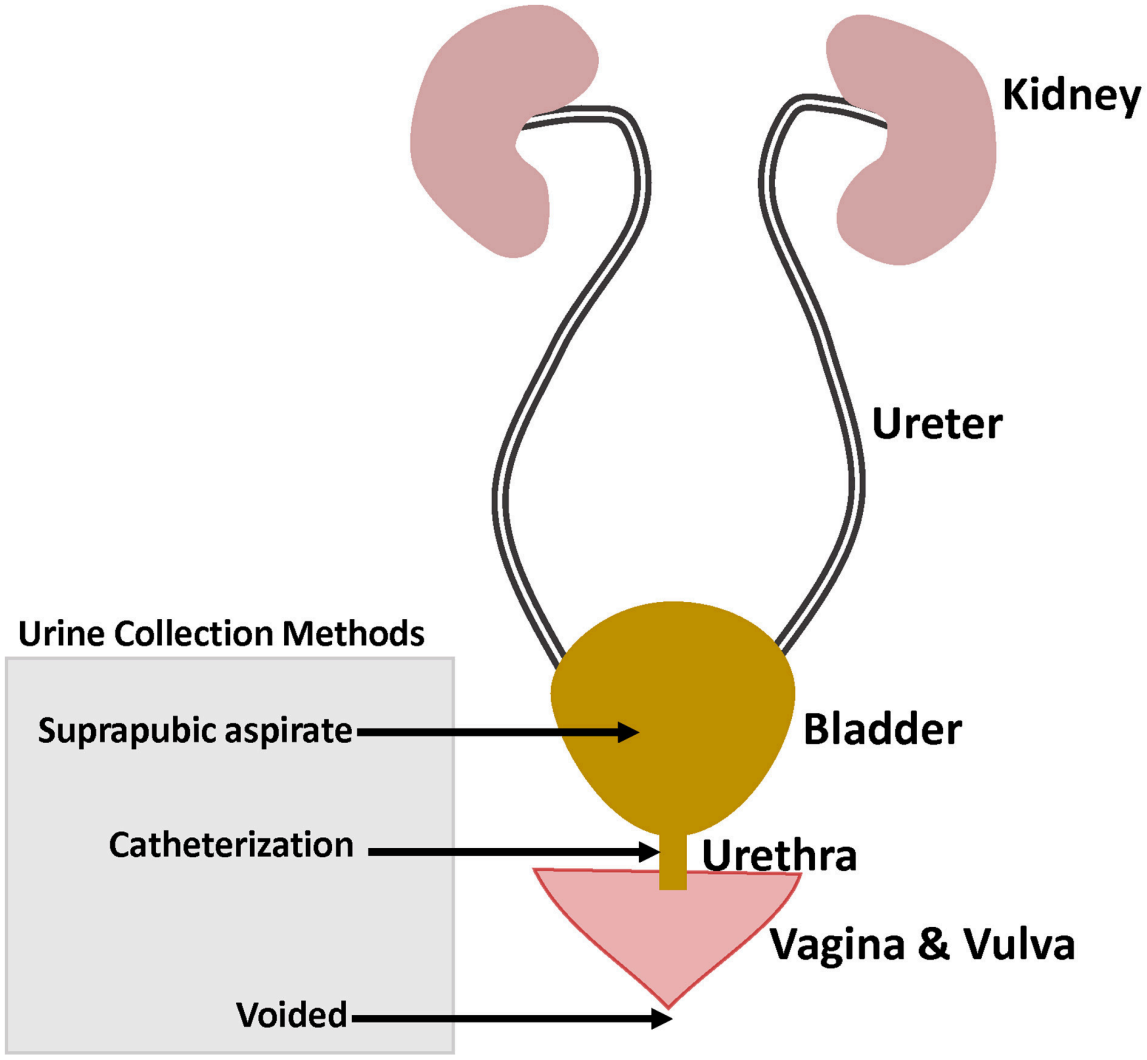
Female genitourinary tract system highlighting the different urine collection methods used in urinary microbiome studies.

The proper interpretation of urobiome studies requires taking into consideration the urine collection methods which included: (1) urination (voided urine), with or without “clean catch” the latter being collection of midstream voided urine after cleaning the skin; (2) transurethral catheter—sampling upwards the urethra; and (3) suprapubic aspiration (SPA) of urine from the bladder (Figure 1). 16S rRNA gene sequencing analysis showed microbiome similarities between catheterized and SPA urine specimens collected from the same patients (Wolfe et al., 2012). However, comparison of voided urine vs. catheterized urine vs. vaginal swabs from same patients showed voided urine contained vulvovaginal bacteria in addition to urinary bacteria (Wolfe et al., 2012).

A considerable diversity of bacterial taxa has been found in catheterized urine from asymptomatic individuals (Hilt et al., 2014; Pearce et al., 2014). Bacterial culture by EQUC and 16S rRNA gene sequencing showed presence of *Lactobacillus, Corynebacterium, Streptococcus, Actinomyces, Staphylococcus, Gardnerella*, and *Bifidobacterium* (Hilt et al., 2014; Pearce et al., 2014).

These recent discoveries raised the question of whether, similarly to other mucosal sites, the urinary bladder harbors resident microbiota that support mucosal tissue integrity, immune barrier, and overall urinary health. Hence, studies have been directed toward characterization of the urinary microbiome and metagenome (the aggregate functional level of the urobiome) associated with urinary health and disease.

## Urinary Microbiome Association With UI Subtypes, Overlapping Syndromes and Therapies

### Microbiome Characteristics by UI Subtypes and Overlapping Syndromes

Urinary microbiome studies using 16S rRNA gene sequencing have shown differences in microbiome profile between different subtypes of UI and asymptomatic individuals. A summary of findings using only catheterized urine samples to establish differences by symptoms or by detection of bacterial DNA shown in Table 1. Below we present these UI studies and studies of the overlapping urinary tract disorders; OAB, IC, and UTIs. We also present comparisons with other methods of urine collection if available within the same studies. These studies support the premise that further urobiome research may lead to new diagnostic criteria that could supplement current diagnostic methods and improve urology subcategorization currently hampered by overlapping symptoms.

**Table 1:** Summary of female urinary incontinence microbiome studies using catheterized urine samples.

**References**	**Study design^a^, ^b^**	**Sample size (# of subjects)**	**UI and reference group**	**Age (years, mean ± SD^c^)**	**BMI^d^ (kg/m^**2**^, mean ± SD^c^)**	**Main findings (UI vs. reference)**
Pearce et al., 2014	Cohort^i^	113	UUI^e^ non-UUI	63 ± 12, 49 ± 14	32 ± 8 28 ±6	↑*Gardnerella* *↓ Lactobacillus*
Pearce et al., 2015	Random, double-blind, controlled trial	179	UUI bacterial DNA sequence positive UUI sequence negative	55.8 ± 12.2 61.3 ± 9	33.7 ± 7.3 30.1 ± 6.6	Eight major bacterial clusters were identified. Seven clusters dominated by a single genus, most commonly *Lactobacillus* (45%) or *Gardnerella* (17%)
Karstens et al., 2016	Case-control	20	UUI non-UUI	57± 8 58 ± 9	31.2 ± 7.8 28.3 ± 6	9/14 bacteria were increased while 5/14 were less abundant. 5/9 increased bacteria were associated with UTIs^f^
Thomas-White et al., 2016	Cohort	134	UUI (subgroups: treatment responders vs. non-responders non-UUI	61.5 ± 11.5 49 ± 14.7	32.7 ± 8.4 28 ± 5.5	Microbiome more diverse in UUI. Treatment non-responders had greater bacterial diversity than responders
Komesu et al., 2018	Multi-site observational	207	MUI^g^ non-MUI	53 ±10.8 53 ±11.7	32.7 ± 7.2 28.4 ± 6.6	Six bacterial community types identified. In women < 51 years, bacterial community types distinguished MUI
Fok et al., 2018	Cohort	126	SUI^h^ bacterial DNA sequence positive SUI sequence negative	57 60	28 27	Two specific bacteria species; *Atopobium vaginae* and *Finegoldia magna* were associated with urinary symptom severity

a 16S rRNA gene sequencing was used to characterize the urinary microbiome.

b a Combination of pre- and post-menopausal women were included.

c Standard deviation.

d Body mass index.

e Urgency urinary incontinence.

f Urinary tract infections.

g Mixed urinary incontinence.

h Stress urinary incontinence.

i EQUC with MALDI-TOF mass spectrometry used to characterize the urinary microbiome.

#### UUI

Two separate studies of catheterized urine from women with and without UUI showed differences in their urinary microbiome signatures (Pearce et al., 2014; Karstens et al., 2016). A case-control study (*n* = 20) using 16S rRNA analysis showed polymicrobial communities in urine from both UUI cases and asymptomatic controls (Karstens et al., 2016). However, the relative abundance of 14 bacteria significantly differed between control and UUI samples. In addition, an increase in UUI symptom severity was moderately correlated with a decrease in microbial diversity.

In another study (*n* = 113), compared to asymptomatic women, urinary microbiome in women with UUI had increased *Gardnerella* and decreased *Lactobacillus* genus abundance (Pearce et al., 2014). Furthermore, species-level comparison of cultured microbiota by EQUC with MALDI-TOF mass spectrometry showed *L. gasseri* was detected more frequently in UUI while *L*. *crispatus* was more frequent in asymptomatic controls. The biological mechanism underlying these species-level associations within the *Lactobacillus* genus is unknown. They may indicate different roles of *L. gasseri* and *L. crispatus* in maintaining a healthy urinary environment. However, the predominant detection of *L. gasseri* in UUI and *L. crispatus* in the absence of UUI could also represent an epiphenomenon reflecting possible *L. gasseri* overgrowth as an imbalanced response to the depletion of *L. crispatus* and other *Lactobacillus* species caused by exposures associated with UI. For example, it has been shown that epithelial colonization by *L. crispatus* is more vulnerable to depletion by urogenital protozoan pathogens compared to *L. gasseri*, and such pathogens would not be detected by standard microbiome analysis using 16S rRNA gene sequencing (Yamamoto et al., 2013). Thus, causality of epidemiologic associations should be explored in experimental bladder models.

#### OAB

Although data from studies using voided urine should be interpreted with caution, analysis of midstream urine from a case-control study of women (*n* = 95) with symptoms of OAB, of whom majority had overlapping UUI (68%), revealed *Lactobacillus* DNA was less prevalent in women with OAB compared to asymptomatic controls (Curtiss et al., 2017). This study also found that *Proteus* DNA, a genus with many uropathogenic species (Drzewiecka, 2016), was more prevalent in women with OAB compared to asymptomatic controls (Curtiss et al., 2017).

#### IC

A recent study by Abernethy et al. suggested that the microbiome may play a role in IC (Abernethy et al., 2017). In this study, 16S rRNA analysis determined the microbiome of catheterized urine from women (*n* = 40) with IC was not dominated by a single genus and was less likely to contain *Lactobacillus* compared to asymptomatic women. Abernethy et al. also showed that *L. acidophilus* was associated with less severe scores on the IC symptoms index (Abernethy et al., 2017). *L. acidophilus* is known to alleviate inflammation (Foye et al., 2012; Li et al., 2016) and is marketed as a probiotic. These findings offer additional evidence in support of the importance of certain *Lactobacillus* species in the healthy bladder milieu.

#### SUI

In one study of women undergoing surgery for SUI, urine specimens were collected from UTI-negative patients and analyzed by 16S rRNA gene sequencing. Of the 197 urine samples, 174 were collected by clean-catch, while only 23—by catheterization. Although 86% of samples had detectable bacterial DNA, no associations were found between bacterial diversity and SUI symptoms (Thomas-White et al., 2017). More recently, a study which included catheterized urine from a mix of SUI and pelvic organ prolapse patients (*n* = 126) showed that two bacterial species; *Atopobium vaginae* and *Finegoldia magna* were associated with preoperative urinary symptom severity (Fok et al., 2018). It is important to note that *A. vaginae* is part of the microbiome signature of bacterial vaginosis (the syndrome of disturbed vaginal microbiota) and may be associated with the urinary symptoms of dysuria reported by these women (Onderdonk et al., 2016).

#### MUI

Using voided and catheterized urine samples, Thomas-White et al. showed that in women with SUI, co-occurrence of UUI symptoms was associated with loss of *Lactobacillus* dominance (Thomas-White et al., 2017). However, a mixed UI study (*n* = 207) using only catheterized urine samples showed overall *Lactobacillus* predominance did not differ between MUI cases and asymptomatic controls (Komesu et al., 2018).

#### UTI

It has been suggested that UI can confer higher risk to developing a UTI (Kow et al., 2016). An earlier study (*n* = 213) that used standard culture methodology on catheterized urine showed that any bacteriuria at ≥10^3^ CFU/ml was more prevalent in incontinent compared to continent women (Walsh et al., 2011). Using more sensitive methodology (enhanced culture and sequencing), recent studies using catheterized urine have shown that women with UUI had increased abundance of bacteria associated with UTIs (Pearce et al., 2014; Karstens et al., 2016). Specifically, EQUC showed that *Actinotignum* [formerly *Actinobaculum* (Yassin et al., 2015)], *Aerococcus, Oligella*, and *Arthrobacter*, which have previously been implicated in UTIs (Funke et al., 1998; Kline and Lewis, 2016; Pagotto et al., 2016), were more frequently cultured from women with UUI than controls (Pearce et al., 2014). Similarly, in a smaller case-control study, women with UUI had increased abundance of 9 bacteria, of which 5 (*Brevundimonas, Alteromonadaceace, Elizabethkingia, Methylobacterium*, and *Stenotrophomonas*) have previously been associated with UTIs (Karstens et al., 2016). However, it is unknown whether these women went on to develop a UTI. Also, it is unclear whether the urinary microbiome in UI promotes colonization by uropathogens (some of them may be emerging and yet unknown) and then in turn these uropathogens change the environment to facilitate further microbiome disturbance. To answer these questions, large longitudinal studies designed to characterize the urinary microbiome in continent and incontinent women with and without UTIs are needed.

## The Influence of the Urinary Microbiome on UI Treatment Outcomes

Oral anticholinergics are used as the primary pharmacotherapy option for UUI and OAB (Geoffrion, 2012). However, some patients do not respond to anticholinergic therapy (Luo et al., 2012). Understanding the biological mechanism for the lack of response to therapy may give insight into how to improve treatment strategies for UUI and OAB. Urinary microbiome has been shown to differ depending on the response to anticholinergic treatment (Thomas-White et al., 2016). Urinary microbiome profiles were characterized using catheterized urine from 74 UUI patients before and after anticholinergic (solifenacin) treatment. Women with UUI were categorized into three treatment response groups after a 12-week period: (1) 5 mg responder, (2) 10 mg responder, and (3) non-responder. Overall treatment increased prevalence of *Lactobacillus* (16S rRNA gene sequenced urotypes before vs. after treatment: 40.5vs. 47.8%). This trend was simultaneously noted by EQUC for *Lactobacillus* (22 vs. 31.6%). When stratified by treatment response, there were significant differences in cultivable bacterial diversity with 5 mg responders having less diversity than 10 mg responders and non-responders. *Actinomyces* and multiple *Streptococcus* species (*S. anginosus* and *S. pneumoniae/mitis/oralis*) were more frequently detected in 10 mg responders while *Corynebacterium, Actinomyces neuii*, and *Staphylococcus simulans* were more common in non-responders compared to 5 mg responders. These findings suggest that the microbial composition of urine and especially bacterial diversity may play a role in anticholinergic treatment response.

## Risk Factors for UI that Influence the Urinary Microbiome

### Sex Steroid Hormones

Estrogen is known to play an important role in the function of the lower urinary tract with estrogen receptors present throughout the urogenital tract (Iosif et al., 1981; Skala et al., 2010, 2011). However, the role of estrogen in UI is unclear. Earlier studies suggested that the increasing prevalence of UI in aging women was linked to declining levels of estrogen that occur during menopausal transition (Iosif and Bekassy, 1984). Thus, estrogen therapy was used for treatment of UI. More recent studies found that UI in middle-aged women is related to higher serum estradiol levels (Teleman et al., 2009) and women with a sharp decline in estradiol levels have significantly lower UI symptom scores (Gopal et al., 2008).

Epidemiological studies investigating hormone therapy in treatment of UI generated mixed results (Grodstein et al., 2004; Hendrix et al., 2005; Iliadou et al., 2009; Townsend et al., 2009). In the Nurses' Health Study, risk of UI was elevated among postmenopausal women on estrogen only, and estrogen combined with synthetic progesterone formulations of hormone replacement therapy (HRT) (Grodstein et al., 2004). Similarly, analysis of postmenopausal women in the Women's Health Initiative randomized clinical trial showed that HRT was associated with increased UI incidence and worsening UI symptoms (Hendrix et al., 2005). In premenopausal women, results from the Swedish Twin Register showed that combined oral contraceptives were associated with reduced risk of symptoms for SUI, UUI, and MUI (Iliadou et al., 2009). Whereas results from the Nurses' Health Study II showed that oral contraceptive use was associated with a modest risk of UI incidence and odds of UI increased with duration of oral contraceptive use (Townsend et al., 2009).

The mechanism of hormone action in UI is not well understood. Exogenous estrogen has been suggested to increase urethral cellular maturation, urethral blood flow, urethral pressure, and modify vaginal flora which may be important for continence (Raz and Stamm, 1993; Waetjen et al., 2011). It is well established that sex hormones play a role in regulating vaginal and intestinal microbiota and in turn microbiota may modify mucosal estrogen levels (Molander et al., 1990; Heinemann and Reid, 2005; Wilson et al., 2007; Brotman et al., 2014; Romero et al., 2014; Chen and Madak-Erdogan, 2016; Shen et al., 2016). Whether the microbiome-hormone cross-talk also occurs in the urinary tract and whether estrogen and progesterone affect the urinary microbiome similarly to the vaginal microbiome needs to be further explored. Promisingly, results from the cross-sectional study by Thomas-White et al. using 174 voided and 23 catheterized urine samples suggested urine microbial diversity is associated with hormonal status (Thomas-White et al., 2017). The urinary microbiome of postmenopausal women not on HRT had higher diversity than premenopausal women (Thomas-White et al., 2017). In addition, postmenopausal women not on HRT had a lower frequency of *Lactobacillus* or *Gardnerella* compared to postmenopausal women on HRT and premenopausal women (Thomas-White et al., 2017). It would be important to confirm these findings in a prospective study designed to investigate the effects of estrogen and/or progesterone on UI symptoms and the urinary microbiome using only catheterized urine samples.

### Age

Studies have shown differences in the urinary microbiome of women by age. In the Anticholinergic vs. Botulinum Toxin A Comparison (ABC) trial, women tended to have more sequence-positive catheterized urine samples when they were younger (mean = 55.8 years) vs. sequence-negative samples (mean = 61.3 years) (Pearce et al., 2015). In sequence-positive urines, hierarchical clustering revealed seven major urotypes: *Lactobacillus, Gardnerella, Gardnerella/Prevotella, Enterobacteriacae, Staphylococcus, Aerococcus*, and *Bifidobacterium*. A more diverse microbiome was found in older women (mean = 61 years), while younger women were more often *Lactobacillus*-positive (mean = 53.2 years) (Pearce et al., 2015). Similarly, a more recent study showed that urinary microbiomes in catheterized urine grouped into two distinct clusters, based on bacterial compositions at the genus level, were significantly different in age (Thomas-White et al., 2018b). Younger women (mean = 51 years) grouped into the less disperse cluster and were more likely to contain *L. iners* while older women (mean = 59 years) grouped into the more disperse cluster and their urinary microbiomes were enriched in a diverse set of pathogens (Thomas-White et al., 2018b). These age group differences across different studies raise the question of whether the variation in the microbiome by age plays a role in accounting for some of the age-related prevalence estimates of UI and its subtypes.

### Body Mass Index (BMI)

In women with UI, BMI has been linked with urinary microbial diversity. In the SUI study by Thomas-White and colleagues, an increase in BMI was associated with an increase in microbial diversity, specifically an increase in community evenness (distribution of taxa) (Thomas-White et al., 2017). In UUI patients, bacterial DNA sequence-positive subjects had a higher BMI compared to sequence-negative subjects (33.7 ± 7.3 vs. 30.1 ± 6.6 kg/m^2^) (Pearce et al., 2015). The urinary microbiome clustering in sequence-positive subjects revealed that 45% of the samples were dominated by *Lactobacillus*, 17% by *Gardnerella*, 23% by an “other” urotype (dominated by *Gardnerella/Prevotella, Enterobacteriaceae, Staphylococcus, Aerococcus, or Bifidobacterium*), and 13% had a “diverse urotype” with no dominant taxa. The median BMI of subjects with *Lactobacillus, Gardnerella* or “other” urotypes were similar (32.5 vs. 32.5 vs. 32.8 kg/m^2^). In contrast, the subjects with the diverse urotype had the highest median BMI (36 kg/m^2^) (Pearce et al., 2015).

**Figure 2:**
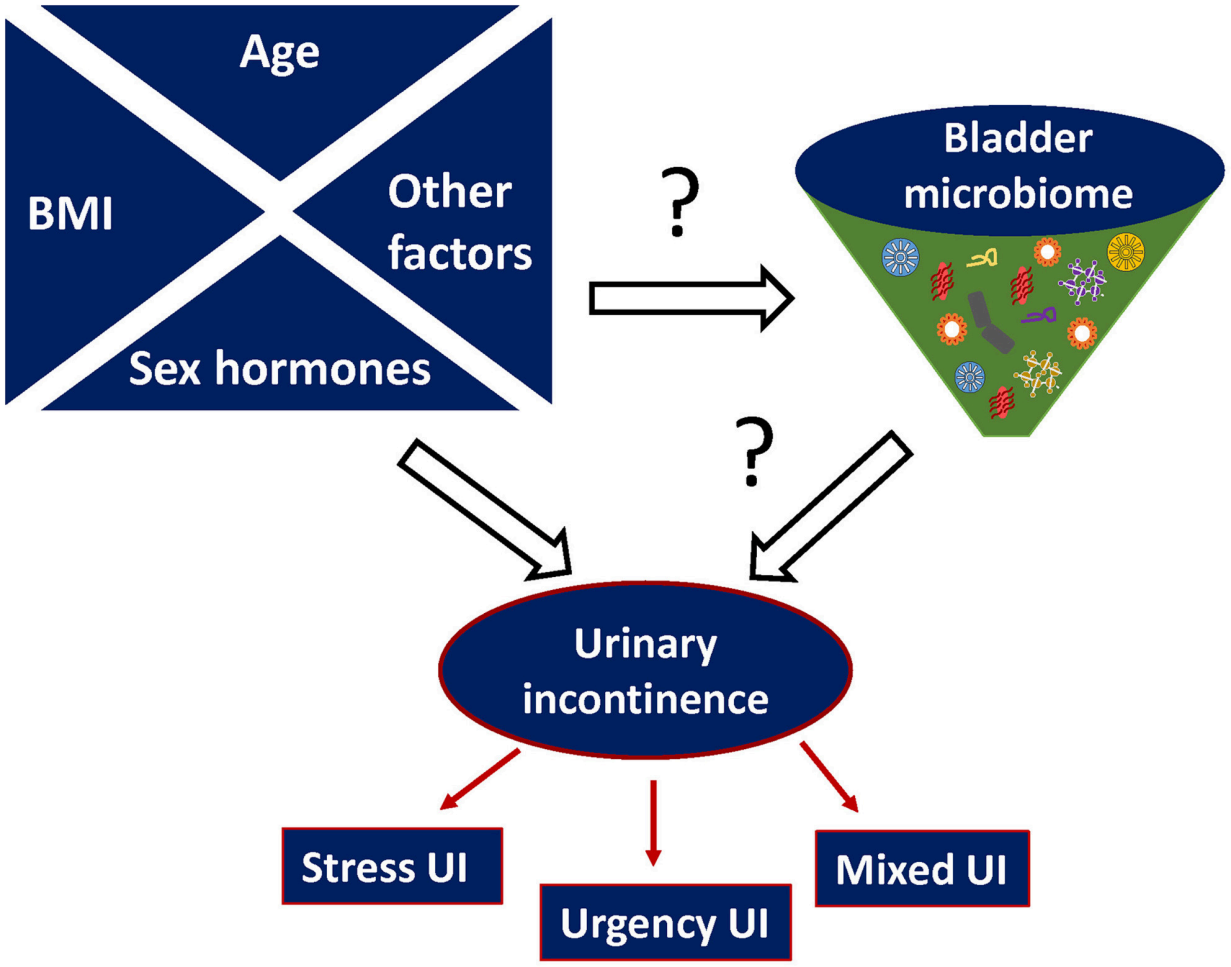
Proposed relationship between urinary microbiome and urinary incontinence (UI). Other possible risk factors that can affect both microbiome imbalance and urinary incontinence include (but are not limited to) hereditary predisposition, metabolic diseases e.g., diabetes, parity etc.

Collectively, evidence suggests there is a relationship between the urinary microbiome and risk factors of UI, such as sex hormones, age, and BMI as illustrated in Figure 2.

## Strategies for Future Urinary Microbial Studies

### The Importance of “-Omics” Technologies and Multi-Omics Approaches

Metagenomic analysis either by 16S rRNA gene or whole genome shotgun sequencing has enabled identification of bacterial communities in multiple anatomical niches such as the oral cavity (Xun et al., 2018), gut (Castaner et al., 2018), genital tract (Ravel et al., 2011; Zozaya et al., 2016), and now the bladder (Fouts et al., 2012; Wolfe et al., 2012; Pearce et al., 2014, 2015). Gut and vaginal microbial studies have also succeeded using transcriptomics (Garrido et al., 2015; Deng et al., 2018), proteomics (Lee et al., 2017; Pinto et al., 2017), and metabolomics (Srinivasan et al., 2015; Vitali et al., 2015; Lamichhane et al., 2018) to unveil functional aspects of the microbiota at these mucosal sites. Transcriptomics and proteomics allow us to functionally characterize active sets of genes and proteins expressed by microbiota and host while metabolomics is a useful tool to understand the complex metabolic interactions between microbe and host (Lamichhane et al., 2018). So far, the majority of urinary incontinence studies have followed an omics approach which either focuses on microbiome characterization (16S rRNA gene sequencing and/or EQUC with MALDI-TOF mass spectrometry) (Table 1) or proteomics to identify protein patterns associated with incontinence (Koch et al., 2016). The combination of microbiome characterization and function approaches may provide greater understanding of the role of the urinary microbiome in health and pathophysiology of disease (Hasin et al., 2017).

### Clinical Relevance of Microbial Community Types, Species-, and Strain-Level Variations

The classification of microbiota into community types has provided a framework for understanding microbial variation in health and disease (Knights et al., 2014). In the gut, three robust microbial clusters termed enterotypes were first identified by Arumugam et al. (2011). These enterotypes were distinguished by enrichment in one of three genera; *Bacteroides, Prevotella*, and *Ruminococcus* (Arumugam et al., 2011). While in the vagina, Ravel et al. identified five community state types (CSTs) (Ravel et al., 2011). Four CSTs (I, II, III, and V) were dominated by *Lactobacillus* specifically; *L. crispatus, L. gasseri, L. iners*, and *L. jensenii* while CST IV termed “diverse” had no specific dominant species. Four cervicotypes (CT) were identified in human uterine cervical secretions—CT1, dominated by *L. crispatus*, CT2, dominated by *L. iners*, CT3—by *Megasphera, Atopobium*, and *Gardnerella*, and CT4—by *Atopobium, Gardnerrela, Prevotella*, and *Fusobacterium* (Anahtar et al., 2015). This simplified classification of complex microbiome data has the potential to serve as an indicator of risk or susceptibility of developing certain conditions. It could also be a useful biomarker to monitor disease progression, and importantly, treatment response (Costea et al., 2018). Importantly, specific microbiome types induce distinct inflammatory signatures proven at the single organism as well as community level and may modify responsiveness to urogenital pathogens (Fichorova et al., 2011; Yamamoto et al., 2013; Anahtar et al., 2015).

For urinary incontinence studies, it would be important to perform similar microbial cluster characterization to identify urinary community types associated with health and disease. Promisingly, a recent study on mixed UI identified six urinary bacterial community types across MUI cases and controls (Komesu et al., 2018). Three types (1, 3, and 6) were dominated by *Lactobacillus* in varying genus abundance ranging from 34.3 to 89.2%. Type 5 was dominated by *Gardnerella* while type 2 and 4 were diverse. Overall, bacterial communities did not differ between all age-matched MUI and controls. However, in women under 51 years, bacterial community types distinguished MUI from controls.

While community types are a convenient way of interpretation by reducing multi-dimensionality, it is important to consider in future studies that species and within species variations at the strain level may also be of critical importance for health and disease. Virulence may vary by strains (Brüggemann et al., 2018). The importance of the strain-level characterization of the microbiome is suggested by several recent studies. For example, such supragingival metagenome analysis identified over 726 strains belonging to 426 species and showed strain-specific associations with dental caries (Al-Hebshi et al., 2019). Higher resolution urinary microbiome analysis tracking microbes at the strain level would also allow asking questions for transmissibility and may contribute to a better understanding of familial predisposition to urinary tract disorders. A large study utilizing the Swedish register has reported that sisters and mothers of women operated for UI/urogenital prolapse had a higher risk of surgery for pelvic floor conditions, and especially sisters of women operated at a young age (<50) and with a low parity (Andrada Hamer and Persson, 2013). The study attributed the finding to heredity; however, we hypothesize that in addition to genetic predisposition, heritable/transmissible specific strains of the commensal microbiome might have contributed to such familial clustering of pelvic floor conditions. As metagenome databases continue to grow in complexity and machine learning ability to detect differences at the strain level, sequencing of urinary isolates from multiple patients and depositing these sequences to public domains may contribute to a deeper understanding of microbiome-driven hereditary risk factors of UI.

Another factor to consider is the contribution of bacteriophages (viruses that infect bacteria) to the urinary microbiome. Recently it has been suggested that bacteriophages within the bladder may contribute urinary tract healthy (Miller-Ensminger et al., 2018). This study analyzed the presence of bacteriophage sequences in 181 bacterial isolates from the bladder of women with and without lower urinary symptoms. They showed variation in the abundances of bacteriophages between bacteria isolated from asymptomatic women and women with OAB.

### Restoring Urinary Tract Health Through Live Biotherapeutics

Evidence on the relationship between the gut and urinary microbiota is mixed. Recently, an interesting observation was made in patients undergoing fecal microbiota transplant (FMT). One year after FMT, the number of recurrent UTIs decreased from 4 to 1 while in the control group without FMT, the number of recurrent UTIs per year did not change (Tariq et al., 2017). This finding suggests a possible relationship between the gut microflora and the bladder microbial communities. However, recent comparative microbiome analysis has shown no connection between the intestinal and bladder sites across healthy women (Thomas-White et al., 2018a). However, a major limitation of this study was that analysis was performed on unpaired samples (Thomas-White et al., 2018a). Thus, it would be important to perform comparative microbiome analysis between these two niches in paired samples within individuals, and especially in specific patient populations such as patients with; recurrent UTI's, neurologic conditions, or the elderly. Similarly, to the success of FMT in treating certain gastro-intestinal conditions, it is plausible to hypothesize that urinary microbiota transplant modalities may be developed in the future to treat women with chronic or persistent urinary conditions such as recurrent UTI's, refractory UUI, and IC.

Evidence suggests that the vagina and urinary microbiota are interconnected (Thomas-White et al., 2018a). Thomas-White et al. compared the microbiomes of catheterized urine samples and vaginal swabs using whole genome sequencing and showed highly similar species reside in bladder and vagina of individual women (Thomas-White et al., 2018a). Therefore, another possible live biotherapeutic approach to restoring the healthy urobiome would be the introduction of specific singular probiotic strain or a selected mix of strains. Probiotics composed of *Lactobacillus* species have been widely used to promote women's vaginal health with variable success (Reid et al., 2001, 2003; Ya et al., 2010; De Alberti et al., 2015; Verdenelli et al., 2016; Russo et al., 2018). However, their efficacy has not yet been confirmed for bladder health. A recent Cochrane Database review by Schwenger et al. showed no difference between placebo and probiotics in reducing recurrent UTIs (Schwenger et al., 2015). Due to the limited number of studies (*n* = 9), which only involved a cumulative total of 735 participants, the review could not reliably establish a beneficial effect. Nevertheless, the emerging data on bladder-colonizing bacteria suggest that bladder-targeted probiotics may become the next therapeutic agents in bladder health. Currently, there is an ongoing study in Australia on the effectiveness of combination oral probiotic therapy with *Lactobacillus reuteri* RC-14+, *Lactobacillus rhamnosus* GR-1 [RC14-GR1 capsules], and/or *Lactobacillus rhamnosus* GG+, *Bifidobacterium* BB-12 in preventing UTI in people with spinal cord injury compared to placebo (Lee et al., 2016).

### Monitoring Microbial Colonization in Infants

The gut is the most extensively studied niche of the human microbiome. Multiple studies have characterized the initial gut microbiota development in infants (Bazanella et al., 2017; Gabriel et al., 2017; Hill et al., 2017). However, no studies have investigated bladder colonization in early life. If we acknowledge that the bladder mucosa physiologically harbors bacteria (Hilt et al., 2014), future studies should monitor bladder colonization in healthy newborns and infants. Such studies will inform us on relations within the urinary microbiome that we cannot currently explain. Collecting, storing, and subsequent microbiome testing of the urine from children may play an essential role in understanding the etiology and onset of urinary disease conditions later in adulthood that are still enigmatic.

## Outlook

Our knowledge of the urinary microbiome is growing fast but much remains to be uncovered. Observational epidemiologic studies should be supplemented by more experimental and interventional studies to investigate causality beyond associations. Well-designed clinical trials controlling for technical as well as biological variation related to longitudinal sampling and confounders may generate microbiome signatures of differential diagnostic value to distinguish UI from other urinary disorders with overlapping symptoms. The beneficial roles of certain *Lactobacillus* species and other urinary commensals should be further investigated to feed a pipeline of live biotherapeutics that may ameliorate drug-resistant UI symptoms. The deeper understanding of the role of the female urobiome in the context of reproductive hormones and its interaction with the host in health and in disease would enable the development of targeted prevention and intervention strategies to resolve UI and other debilitating urinary tract disorders thus improving women's general health and quality of life.

## Author Contributions

All the authors contributed extensively to the work presented in this manuscript and approved it for publication. The manuscript was drafted by YG and IG and was critically revised by RF and VM.

### Conflict of Interest Statement

The authors declare that the research was conducted in the absence of any commercial or financial relationships that could be construed as a potential conflict of interest.
